# Machine learning versus traditional regression models for predicting diabetic retinopathy screening adherence among community-dwelling older adults with diabetes

**DOI:** 10.3389/fendo.2026.1909594

**Published:** 2026-08-04

**Authors:** Yawen Wang, Lanting Xia, Guiling Geng, Chunxing Ge, Haiyan Shen, Xiangyun Qian

**Affiliations:** 1 School of Nursing and Rehabilitation, Nantong University, Nantong, China; 2 Teaching and Research Section, Nantong College of Science and Technology, Nantong, China; 3 Department of Geriatrics, Nantong Third People’s Hospital (Affiliated Nantong Hospital 3 of Nantong University), Nantong, China; 4 Nursing Department, Nantong Third People’s Hospital (Affiliated Nantong Hospital 3 of Nantong University), Nantong, China

**Keywords:** community-dwelling older adults, diabetic retinopathy screening adherence, logistic regression, machine learning, protection motivation theory, random forest

## Abstract

**Background:**

Diabetic retinopathy (DR) is a leading cause of preventable vision loss among patients with diabetes, yet screening adherence remains suboptimal. Existing studies have mainly focused on clinical or sociodemographic determinants, with limited evidence integrating psychological mechanisms. The application of Protection Motivation Theory (PMT)-based constructs combined with machine learning for predicting DR screening adherence remains underexplored in community populations.

**Objective:**

This study compared machine learning models (decision tree and random forest) with traditional logistic regression for predicting DR screening adherence among community-dwelling older adults with diabetes, incorporating a validated PMT-based questionnaire as a key psychological predictor.

**Methods:**

A cluster random sampling design recruited 1,021 older adults with diabetes from four community health centers in Nantong, China (March–October 2025). Data included sociodemographic characteristics, clinical indicators, health behaviors, and PMT-based constructs. Participants were classified as good (n = 159) or poor adherence (n = 862). SMOTE and class weighting addressed class imbalance. Models were evaluated using 10-fold cross-validation. Logistic regression, decision tree, and random forest models were built using identical predictors. Decision curve analysis assessed clinical utility.

**Results:**

The random forest model achieved a marginally higher AUC (0.774, 95% CI: 0.678–0.869) compared with logistic regression (AUC = 0.751, 95% CI: 0.678–0.824) and decision tree (AUC = 0.709, 95% CI: 0.596–0.821); however, DeLong’s tests indicated no statistically significant differences (all p > 0.05). The decision tree exhibited the best calibration (lowest Brier score = 0.1142). DCA indicated that random forest provided the highest net benefit across most threshold probabilities. Multivariable analysis identified history of ocular disease (OR = 3.529, 95% CI: 2.430–5.139) as the strongest positive predictor, while higher HbA1c, lower self-efficacy, higher perceived severity, absence of exercise therapy, and smoking were associated with poorer adherence.

**Conclusion:**

Machine learning models demonstrated comparable discriminative performance to traditional logistic regression for predicting DR screening adherence, while offering distinct profiles in calibration and clinical net benefit. Integration of PMT-based psychological constructs with clinical and behavioral factors provides a multidimensional framework for understanding screening behavior and supports risk stratification for precision diabetes care.

## 1 Introduction

Diabetic retinopathy (DR) is one of the most common and vision-threatening microvascular complications of diabetes mellitus. Globally, approximately 34.6% of patients with diabetes are affected by DR, with a prevalence of 23.0% in China, the highest in Asia (1). Early fundus screening is essential for preventing vision loss; however, adherence remains suboptimal, with early detection rates in China reported to be below 20% (2), representing a major public health burden.

DR screening adherence is influenced by multiple factors, including sociodemographic characteristics, clinical conditions, health behaviors, and psychological determinants. Older age, lower income, and lower educational level are associated with poorer adherence (3). Clinical factors, including longer diabetes duration, poor glycemic control, and higher comorbidity burden, may further reduce screening participation (4, 5). Intervention studies have demonstrated that reminder systems can improve screening attendance, although socioeconomic disparities remain important barriers (6). In addition, smoking, physical inactivity, and poor dietary behaviors have been identified as predictors of non-adherence (7). These findings indicate that DR screening behavior is determined by multidimensional factors.

Beyond demographic and clinical determinants, psychological factors play an important role in preventive health behaviors. Studies based on the Health Belief Model (HBM) have identified self-efficacy, perceived risk, and disease knowledge as important predictors of screening behavior (8, 9). However, existing evidence has mainly focused on individual psychological constructs, limiting a comprehensive understanding of behavioral mechanisms. Protection Motivation Theory (PMT) provides an integrated framework incorporating threat appraisal (perceived severity and susceptibility) and coping appraisal (self-efficacy and response efficacy), with coping appraisal factors considered important determinants of protective behaviors (10–12). However, PMT-based psychological factors have been less extensively investigated in DR screening behavior. Our team previously developed a PMT-based diabetic retinopathy risk perception questionnaire, which demonstrated good reliability and validity (Cronbach’s α = 0.865; S-CVI = 0.968), enabling systematic assessment of psychological determinants.

Traditional logistic regression is widely used to identify predictors of DR screening adherence; however, its linear assumptions may limit its ability to capture complex nonlinear relationships. Machine learning methods have increasingly been applied in clinical prediction and healthcare decision support. Recent advances in artificial intelligence, including deep learning and explainable AI approaches, have shown potential for extracting complex patterns from clinical data and improving personalized healthcare decision-making (13–16). However, most existing AI applications have focused on disease diagnosis, medical image analysis, and clinical decision support, whereas their application in predicting preventive health behaviors remains relatively limited. Although previous studies have compared machine learning and logistic regression models for DR risk prediction (17, 18), these studies mainly focused on disease occurrence rather than screening behavior and did not incorporate PMT-based psychological variables.

Importantly, no study has simultaneously compared logistic regression, decision tree, and random forest models for predicting DR screening adherence while incorporating PMT-based psychological variables. Existing research has largely focused on HBM frameworks or single-factor analyses, with limited exploration of multidimensional determinants.

Therefore, this study aimed to compare logistic regression, decision tree, and random forest models for predicting DR screening adherence among community-dwelling older adults with diabetes and to evaluate the contribution of PMT-based psychological factors. This multidimensional approach may facilitate early identification of individuals at risk of poor adherence and support targeted intervention strategies in community diabetes care.

## 2 Methods

### 2.1 Study population

A cluster random sampling method was used. From March to October 2025, four community health service centers participating in elderly health examinations in Nantong City (including Tangzha and Xiaohai communities) were randomly selected. Older adults with diabetes who met the eligibility criteria were invited to participate in a questionnaire survey.

Inclusion criteria:

1. aged ≥60 years and diagnosed with diabetes according to standard diagnostic criteria;
2. permanent community residents with residence duration ≥6 months;
3. ability to communicate and complete questionnaires independently;
4. willingness to participate and provide written informed consent.

Exclusion criteria:

1. ocular conditions interfering with fundus examination (e.g., severe cataract with visual acuity <0.1 without surgical treatment, corneal opacity, or eyelid closure disorders);
2. psychiatric disorders, cognitive impairment, or severe comorbid conditions;
3. participation in other concurrent studies.

The study was approved by the Ethics Committee of the Third People’s Hospital Affiliated with Nantong University (Approval No. EK2024055). Written informed consent was obtained from all participants.

### 2.2 Data collection and study variables

#### 2.2.1 Sociodemographic, clinical, and behavioral variables

A structured questionnaire was used to collect sociodemographic characteristics, disease-related information, and health behaviors.Sociodemographic variables included age, sex, education level, marital status, and religious belief. Clinical variables included diabetes duration, family history, treatment modality, laboratory indicators (HbA1c, lipids, renal function), and history of ocular disease. Behavioral variables included smoking status, alcohol consumption, physical activity, sleep duration, and dietary habits.

#### 2.2.2 PMT-based diabetic retinopathy risk perception questionnaire

Psychological variables were assessed using a Protection Motivation Theory (PMT)-based diabetic retinopathy risk perception questionnaire, which was previously developed and validated among patients with diabetes. The questionnaire consists of 30 items across seven dimensions: perceived severity, perceived susceptibility, internal rewards, external rewards, self-efficacy, response efficacy, and response cost. Each item was rated on a 5-point Likert scale (1 = strongly disagree to 5 = strongly agree). Items related to internal rewards, external rewards, and response cost were reverse scored, with higher scores indicating stronger protective motivation-related perceptions toward diabetic retinopathy prevention. The questionnaire demonstrated satisfactory reliability and validity (overall Cronbach’s α = 0.865; scale-level content validity index = 0.968). The complete questionnaire items with corresponding English translations are provided in **Supplementary Table 1**.

#### 2.2.3 Quality control

Data collection followed standardized procedures. Trained investigators conducted face-to-face one-to-one questionnaire administration, provided uniform instructions, and checked completed questionnaires for completeness and logical consistency. Missing or unclear responses were verified on site, and investigator-assisted responses were confirmed with participants. All questionnaires were independently reviewed by two investigators, followed by parallel data entry and cross-verification to ensure data accuracy.

### 2.3 Outcome definition

The primary outcome was maintenance of diabetic retinopathy (DR) screening behavior. Current guidelines recommend at least annual fundus examinations for individuals with diabetes, with more frequent examinations for patients with established DR according to disease severity and clinical judgment (19, 20). However, adherence to these recommendations remains suboptimal in real-world settings (8, 21, 22).

Based on the adherence continuum framework for infrequent but repeated health behaviors (23), good adherence was defined as having at least two fundus examinations within the previous 12 months. This definition was selected to reflect sustained screening behavior rather than a single episode of attendance, which may represent reactive or symptom-driven care (24, 25). Participants with fewer than two examinations were classified as having poor adherence.

A sensitivity analysis was conducted using the guideline-recommended threshold of at least one fundus examination within the previous 12 months. The sensitivity analysis included the same 1,021 participants and applied the same six predictors and stratified 10-fold cross-validation procedures, with only the outcome definition modified.The predictive performance remained highly consistent across the two outcome definitions, with a maximum absolute AUC difference of 0.002, supporting the robustness of the study findings (**Supplementary Table 2**).

### 2.4 Sample size estimation

A total of 1,021 participants were included, of whom 159 (15.6%) had good adherence and 862 (84.4%) had poor adherence.For logistic regression, the events-per-variable (EPV) was 26.5 (159/6), exceeding the recommended threshold.Although no formal sample size formula exists for decision tree and random forest models, the sample size combined with stratified 10-fold cross-validation ensured model stability and generalizability.

### 2.5 Statistical analysis and model development

Analyses were performed using R software (version 4.5.1). The main packages included stats, caret, rpart, ranger, pROC, and rmda. Missing data were assessed before analysis. Of the 1,070 recruited participants, 49 (4.6%) with incomplete information were excluded, resulting in a final analytical sample of 1,021 participants using complete-case analysis. The excluded participants did not differ significantly from those included in the analysis in key sociodemographic and clinical characteristics (all P > 0.05).

Continuous variables were expressed as mean ± standard deviation and compared using independent-sample t-tests, while categorical variables were presented as frequencies and percentages and compared using chi-square or Fisher’s exact tests, as appropriate. Univariable logistic regression was performed to identify candidate predictors, followed by multivariable logistic regression with backward stepwise selection based on the Akaike information criterion (AIC). The six retained predictors were used as inputs for logistic regression, decision tree, and random forest models to ensure a consistent predictor framework and fair performance comparison. No additional algorithm-specific feature selection was applied to maintain comparability across algorithms.

Interaction terms between PMT constructs and clinical variables were not included because the primary objective was predictive model development rather than effect modification analysis, and additional interaction terms could increase model complexity given the limited number of positive adherence events (n = 159).

#### 2.5.1 Logistic regression model

A multivariable logistic regression model was constructed. Odds ratios (ORs) and 95% confidence intervals (CIs) were reported. Forest plots were used to visualize both univariable and multivariable results.Potential effect modification between PMT constructs and clinical variables was not explored in the primary analysis.

#### 2.5.2 Decision tree and random forest models

To address class imbalance between the poor and good adherence groups (1:5.4), SMOTE and class weighting were applied within each training fold during cross-validation. SMOTE generated synthetic samples for the minority class, whereas class weighting increased the penalty for misclassification of minority-class observations. Although advanced oversampling approaches, such as SMOTEDNN, have been proposed for more complex classification scenarios (26), traditional SMOTE combined with class weighting was adopted in this study considering the moderate imbalance ratio, sample size, and the need for clinically interpretable models. Four imbalance-handling strategies (no handling, SMOTE alone, class weighting alone, and SMOTE combined with class weighting) were compared (**Supplementary Figure 1**; **Supplementary Table 3**).

All imbalance-handling procedures were conducted exclusively within the training folds, while validation folds remained independent to prevent data leakage. Decision tree models were developed using recursive binary splitting, and random forest models were constructed using bootstrap aggregation of multiple decision trees. Hyperparameters were optimized within the training folds using grid search. The decision tree complexity parameter (cp: 0.001–0.05) and random forest parameters, including the number of trees (100–1000), mtry (1–6), and minimum node size (5–20), were tuned. The optimal parameter combinations were selected based on the highest mean AUC during stratified 10-fold cross-validation, with AUC specified as the optimization metric. The optimized models were subsequently evaluated using the same stratified 10-fold cross-validation framework.

#### 2.5.3 Model evaluation

Model performance was assessed using receiver operating characteristic (ROC) analysis, with AUC, sensitivity, specificity, and Youden index reported. Pairwise comparisons of AUCs among the three models were performed using DeLong’s test as an exploratory analysis. Given that model performance was evaluated using stratified 10-fold cross-validation, the results of DeLong’s test were interpreted cautiously. The Brier score was calculated to evaluate probabilistic calibration. Decision curve analysis (DCA) was used to assess clinical net benefit across different threshold probabilities. Calibration curves with LOESS smoothing were generated to evaluate the agreement between predicted probabilities and observed outcomes.

## 3 Results

### 3.1 Participant characteristics and univariate analysis

A total of 1,021 community-dwelling older adults with diabetes were included in this study, of whom 159 (15.6%) demonstrated good adherence to diabetic retinopathy (DR) screening and 862 (84.4%) demonstrated poor adherence.The overall mean age of participants was 72.3 ± 6.3 years, and approximately half were female. No significant differences were observed between the two groups in age or sex distribution, indicating a relatively comparable demographic structure.

**Figure 1:**
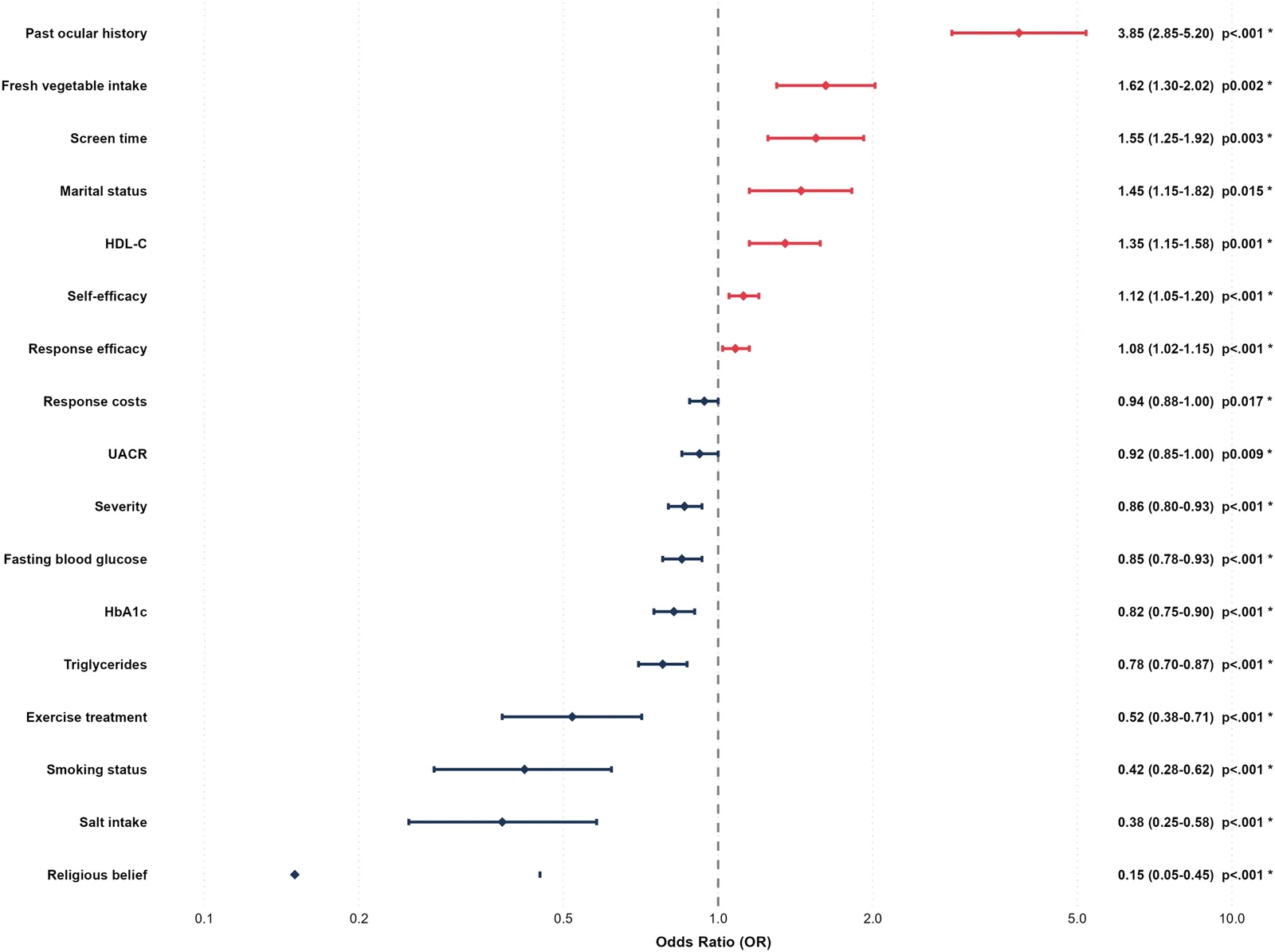
Forest plot of univariable logistic regression analysis.

Significant between-group differences were observed in HbA1c, HDL-C, urinary albumin-to-creatinine ratio (UACR), perceived severity, self-efficacy, exercise therapy, smoking status, history of ocular disease, and other variables (all P < 0.05) (**Table 1**). Univariable logistic regression identified 17 variables significantly associated with DR screening adherence (P < 0.05), and these associations were visualized using forest plots (**Figure 1**).

**Table 1:** Univariate analysis of factors associated with diabetic retinopathy screening adherence.

Variables	Poor Adherence(n = 862)	Good Adherence(n = 159)	p value
Age (years)	72.34 ± 6.29	72.07 ± 6.48	0.747
BMI (kg/m²)	25.55 ± 3.28	25.38 ± 3.59	0.442
Fasting blood glucose (mmol/L)	7.62 ± 2.04	6.90 ± 1.72	<.001
HbA1c (%)	7.49 ± 1.92	6.85 ± 1.62	<.001
Total cholesterol (mmol/L)	4.71 ± 1.21	4.55 ± 1.28	0.103
Triglycerides (mmol/L)	2.03 ± 1.62	1.61 ± 1.18	<.001
LDL-C (mmol/L)	2.64 ± 0.98	2.47 ± 0.96	0.036
HDL-C (mmol/L)	1.33 ± 0.39	1.46 ± 0.45	0.001
UACR (mg/g)	6.78 ± 10.24	5.88 ± 8.26	0.009
DM duration (years)	9.73 ± 6.78	9.84 ± 7.29	0.984
Severity	11.79 ± 2.61	10.28 ± 3.22	<.001
Susceptibility	21.12 ± 5.15	20.15 ± 5.97	0.031
Internal rewards	8.34 ± 2.55	8.28 ± 3.46	0.841
External rewards	8.54 ± 2.24	8.39 ± 3.01	0.665
Self-efficacy	22.67 ± 4.02	20.02 ± 5.59	<.001
Response efficacy	17.69 ± 3.73	16.36 ± 4.47	<.001
Response costs	10.45 ± 2.81	11.04 ± 4.00	0.017
Sex	Male (415, 48.1%)	Male (80, 50.3%)	0.677
	Female (447, 51.9%)	Female (79, 49.7%)	
Religious belief	Yes (137, 15.9%)	Yes (4, 2.5%)	<.001
	No (725, 84.1%)	No (155, 97.5%)	
Residential location	Urban (438, 50.8%)	Urban (67, 42.1%)	0.054
	Rural (424, 49.2%)	Rural (92, 57.9%)	
Marital status	Married (774, 89.8%)	Married (132, 83.0%)	0.015
	Unmarried (6, 0.7%)	Unmarried (1, 0.6%)	
	Divorced (1, 0.1%)	Divorced (2, 1.3%)	
	Widowed (80, 9.3%)	Widowed (24, 15.1%)	
Educational level	Primary or below (507, 58.8%)	Primary or below (78, 49.1%)	0.115
	Junior high (238, 27.6%)	Junior high (52, 32.7%)	
	High school (101, 11.7%)	High school (26, 16.4%)	
	College or above (16, 1.9%)	College or above (3, 1.9%)	
Personal monthly income	≤2000 (362, 42.0%)	≤2000 (57, 35.8%)	0.444
	2001-5000 (384, 44.5%)	2001-5000 (81, 50.9%)	
	5001-10000 (94, 10.9%)	5001-10000 (18, 11.3%)	
	>10000 (22, 2.6%)	>10000 (3, 1.9%)	
Medical insurance type	Self-paid (9, 1.0%)	Self-paid (9, 5.7%)	<.001
	Public funded (1, 0.1%)	Public funded (5, 3.1%)	
	New rural cooperative (403, 46.8%)	New rural cooperative (76, 47.8%)	
	Urban resident (251, 29.1%)	Urban resident (16, 10.1%)	
	Urban employee (198, 23.0%)	Urban employee (53, 33.3%)	
Smoking status	Never (477, 55.3%)	Never (113, 71.1%)	<.001
	Past (271, 31.4%)	Past (25, 15.7%)	
	Current (114, 13.2%)	Current (21, 13.2%)	
Drinking status	Never (461, 53.5%)	Never (95, 59.7%)	0.335
	Past (156, 18.1%)	Past (26, 16.4%)	
	Current (245, 28.4%)	Current (38, 23.9%)	
Screen time	≤2h (259, 30.0%)	≤2h (69, 43.4%)	0.003
	2-4h (362, 42.0%)	2-4h (63, 39.6%)	
	4-6h (203, 23.5%)	4-6h (21, 13.2%)	
	>6h (38, 4.4%)	>6h (6, 3.8%)	
Sleep duration	≤4h (6, 0.7%)	≤4h (2, 1.3%)	0.066
	4-6h (150, 17.4%)	4-6h (40, 25.2%)	
	6-8h (572, 66.4%)	6-8h (90, 56.6%)	
	>8h (134, 15.5%)	>8h (27, 17.0%)	
Exercise frequency	≤1h (197, 22.9%)	≤1h (49, 30.8%)	0.087
	1-2h (550, 63.8%)	1-2h (96, 60.4%)	
	2-4h (94, 10.9%)	2-4h (13, 8.2%)	
	>4h (21, 2.4%)	>4h (1, 0.6%)	
Fresh vegetable intake	<200g (19, 2.2%)	<200g (3, 1.9%)	0.002
	200-400g (177, 20.5%)	200-400g (54, 34.2%)	
	400-600g (392, 45.5%)	400-600g (55, 34.8%)	
	>600g (274, 31.8%)	>600g (46, 29.1%)	
Fresh fruit intake	<200g (432, 50.1%)	<200g (92, 57.9%)	0.170
	200-400g (400, 46.4%)	200-400g (59, 37.1%)	
	400-600g (26, 3.0%)	400-600g (7, 4.4%)	
	>600g (4, 0.5%)	>600g (1, 0.6%)	
Salt intake	<4g (36, 4.2%)	<4g (18, 11.3%)	<.001
	4-6g (630, 73.1%)	4-6g (90, 56.6%)	
	6-8g (177, 20.5%)	6-8g (45, 28.3%)	
	≥8g (19, 2.2%)	≥8g (6, 3.8%)	
Fish intake (weekly)	≤1 time (339, 39.3%)	≤1 time (59, 37.1%)	0.366
	1–2 times (354, 41.1%)	1–2 times (61, 38.4%)	
	>2 times (169, 19.6%)	>2 times (39, 24.5%)	
Family history of diabetes	No (504, 58.5%)	No (98, 61.6%)	0.510
	Yes (358, 41.5%)	Yes (61, 38.4%)	
Type of diabetes mellitus	Type 1 (55, 6.4%)	Type 1 (5, 3.1%)	0.158
	Type 2 (807, 93.6%)	Type 2 (154, 96.9%)	
Diet control	No (101, 11.7%)	No (22, 13.8%)	0.534
	Yes (761, 88.3%)	Yes (137, 86.2%)	
Insulin injection	No (693, 80.4%)	No (133, 83.6%)	0.396
	Yes (169, 19.6%)	Yes (26, 16.4%)	
Exercise treatment	No (217, 25.2%)	No (67, 42.1%)	<.001
	Yes (645, 74.8%)	Yes (92, 57.9%)	
Oral hypoglycemic agents	No (122, 14.2%)	No (25, 15.7%)	0.693
	Yes (740, 85.8%)	Yes (134, 84.3%)	
Past ocular history	No (678, 78.7%)	No (79, 49.7%)	<.001
	Yes (184, 21.3%)	Yes (80, 50.3%)	

continuous variables: mean ± SD; categorical variables: n (%).

### 3.2 Multivariable logistic regression analysis

**Figure 2:**
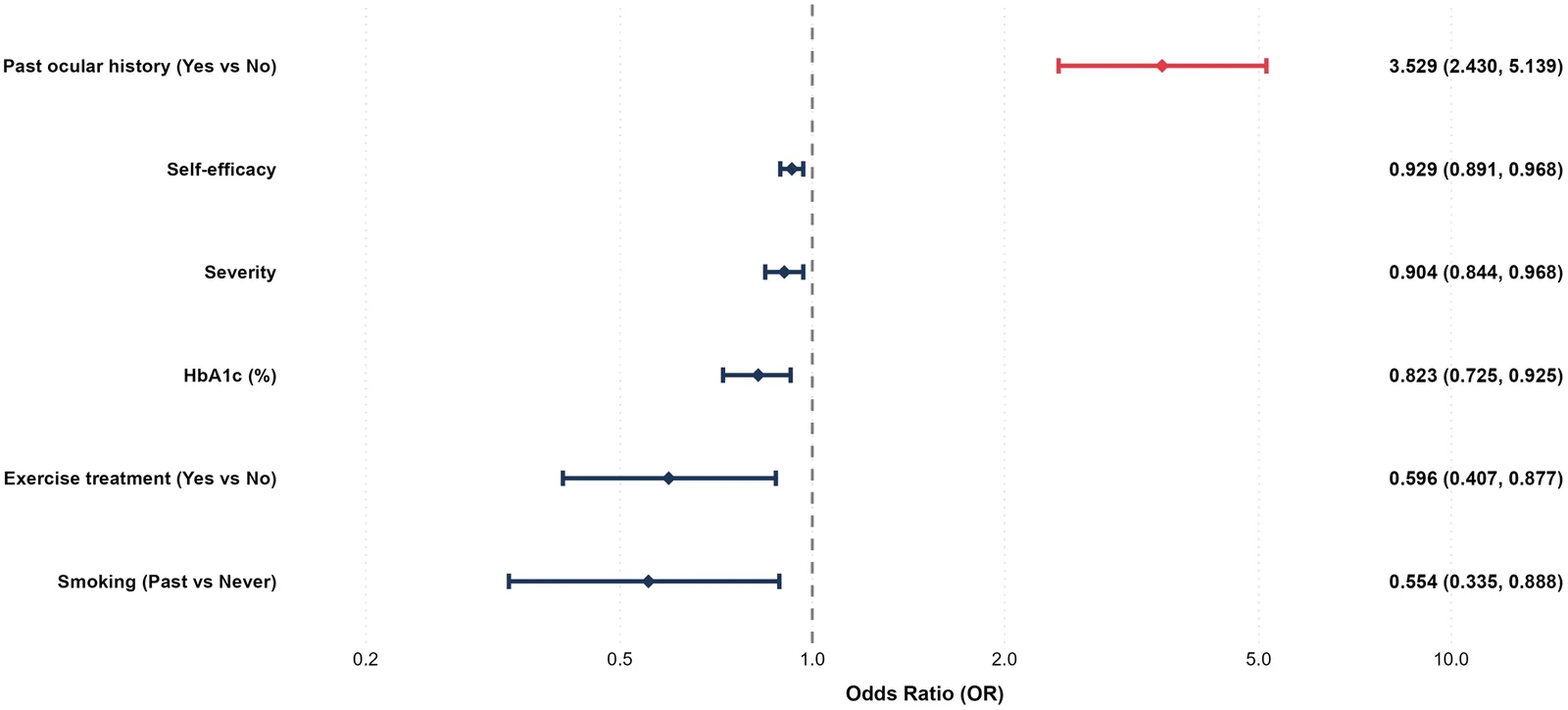
Forest plot of multivariable logistic regression analysis.

After adjustment using multivariable logistic regression with backward stepwise selection based on the Akaike information criterion (AIC), six independent predictors of DR screening adherence remained statistically significant (all P < 0.05) (**Table 2**, **Figure 2**).

**Table 2:** Multivariable analysis of factors associated with diabetic retinopathy screening adherence.

Variable	β	SE	Wald χ²	OR (95% CI)	p value
Self-efficacy	-0.074	0.021	12.115	0.929 (0.891, 0.968)	<.001
Severity	-0.101	0.035	8.292	0.904 (0.844, 0.968)	0.004
HbA1c (%)	-0.194	0.062	9.765	0.823 (0.725, 0.925)	0.002
Exercise treatment (Yes vs No)	-0.517	0.195	6.986	0.596 (0.407, 0.877)	0.008
Smoking (Past vs Never)	-0.590	0.248	5.687	0.554 (0.335, 0.888)	0.017
Past ocular history (Yes vs No)	1.261	0.191	43.683	3.529 (2.430, 5.139)	<.001

OR, odds ratio; CI, confidence interval.

History of ocular disease was positively associated with adherence (OR = 3.529, 95% CI: 2.430–5.139). In contrast, self-efficacy (OR = 0.929, 95% CI: 0.891–0.968), perceived severity (OR = 0.904, 95% CI: 0.844–0.968), HbA1c (OR = 0.823, 95% CI: 0.725–0.925), exercise therapy (OR = 0.596, 95% CI: 0.407–0.877), and smoking status (OR = 0.554, 95% CI: 0.335–0.888) were negatively associated with adherence.

### 3.3 Decision tree structure and random forest variable importance

The decision tree (DT) model identified five key predictors of DR screening adherence: history of ocular disease, self-efficacy, perceived severity, exercise therapy, and HbA1c.

**Figure 3:**
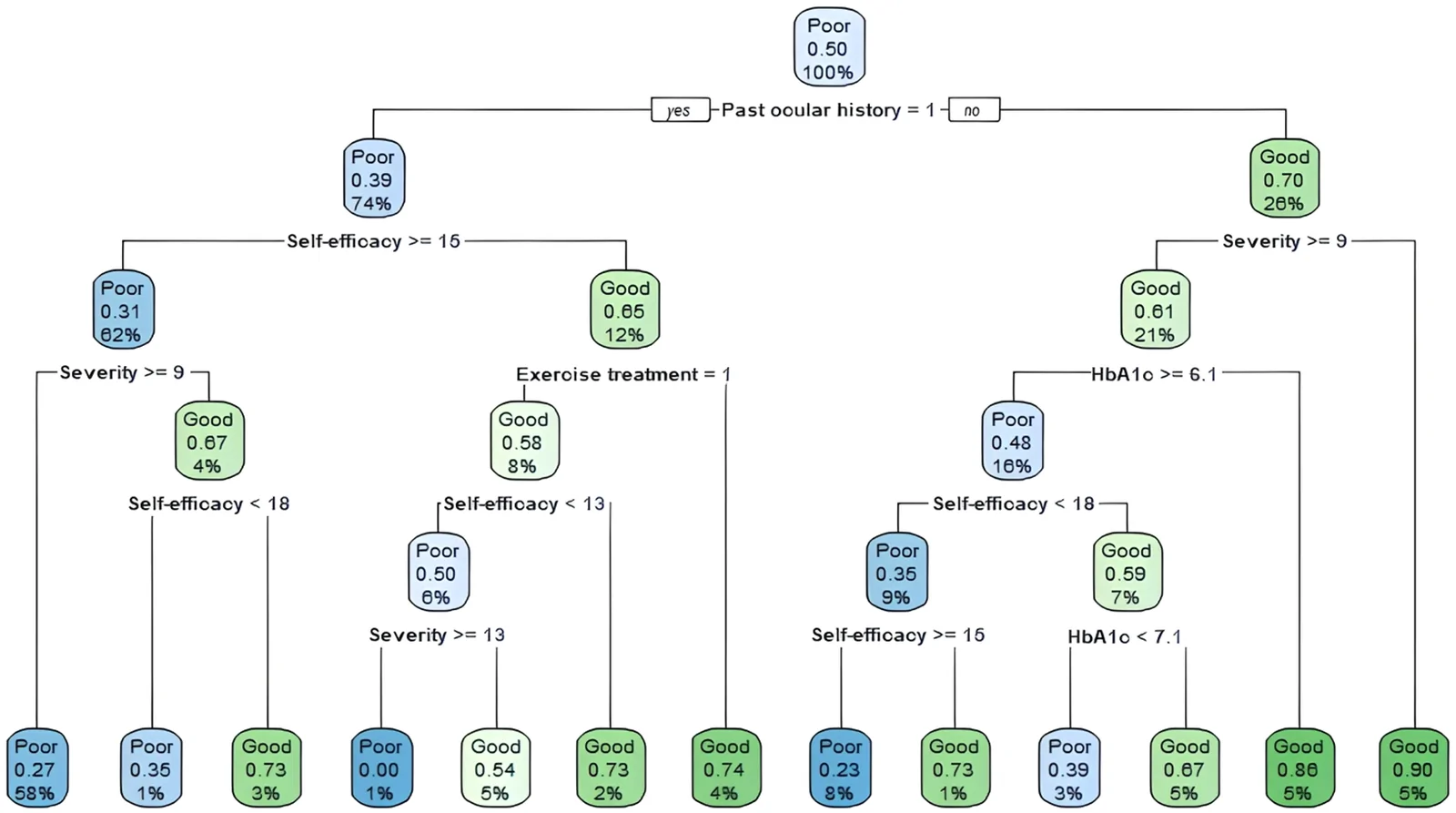
Machine learning decision tree model for fundus examination adherence.

History of ocular disease served as the root node, indicating the strongest initial discriminatory ability. Subsequent splits were based on self-efficacy, perceived severity, exercise therapy, and HbA1c, resulting in a five-level tree structure with 13 terminal nodes (**Figure 3**).

**Figure 4:**
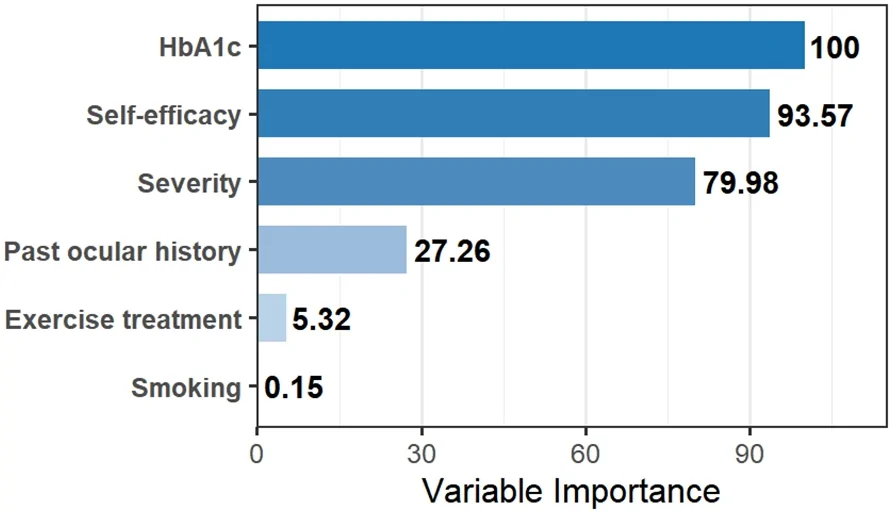
Variable importance for fundus examination adherence prediction.

In the random forest (RF) model, HbA1c ranked as the most important predictor (100.00), followed by self-efficacy (93.57), perceived severity (79.98), history of ocular disease (27.26), exercise therapy (5.32), and smoking status (0.15) (**Figure 4**).

### 3.4 Model performance, validation, and clinical utility

**Figure 5:**
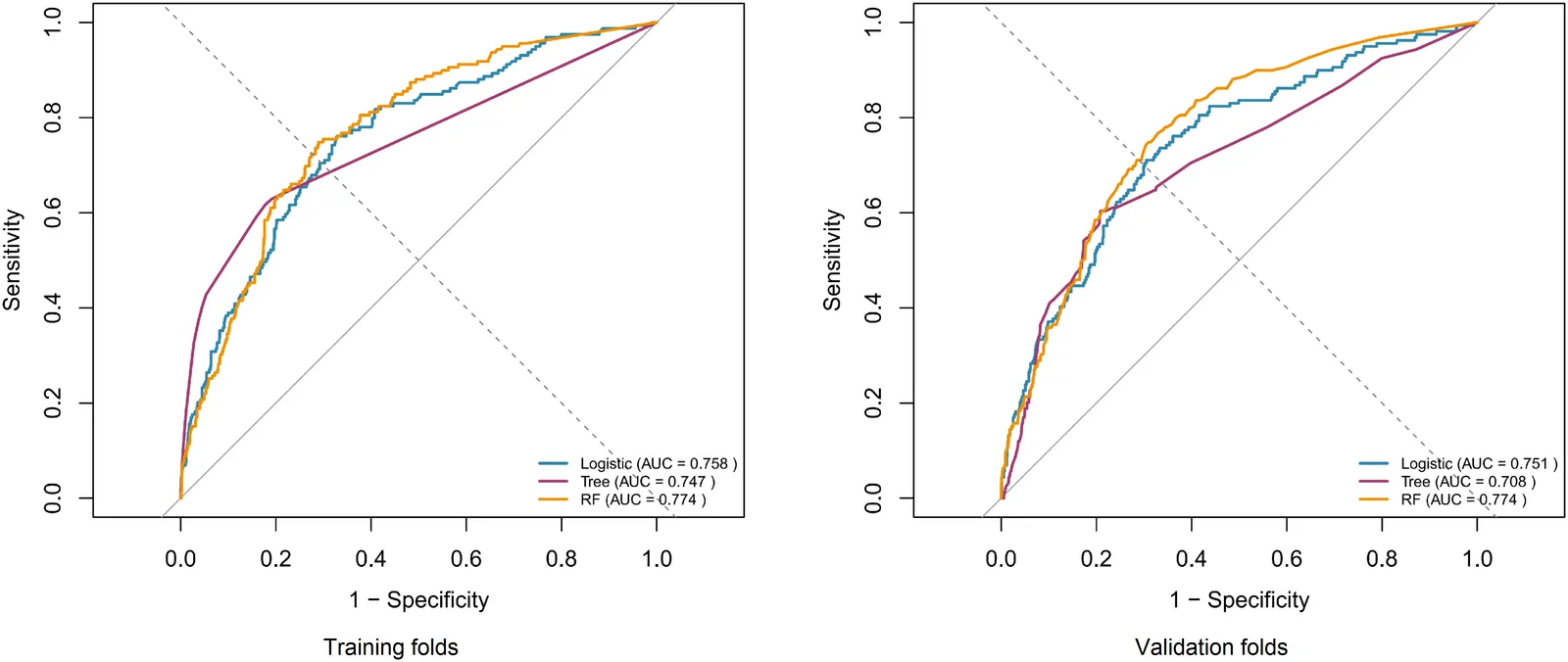
ROC curves of three prediction models based on 10-fold cross-validation. Left panel: training folds; Right panel: validation folds.

Model performance was evaluated using stratified 10-fold cross-validation and ROC analysis (**Figure 5**). In the validation set, the random forest (RF) model showed the highest discriminative performance (AUC = 0.774), followed by logistic regression (AUC = 0.751) and decision tree (AUC = 0.708). The RF model also demonstrated the highest sensitivity (0.748), specificity (0.710), and Youden index (0.458), whereas the logistic regression model showed balanced performance (AUC = 0.751, sensitivity = 0.761, specificity = 0.672). The decision tree model showed higher specificity (0.823) but lower sensitivity (0.616), resulting in a lower AUC (0.709) (**Table 3**).

**Table 3:** ROC-related parameters of three prediction models.

Model	AUC	Standard Error	95%CI	P value	Sensitivity	Specificity	Youden index
Logistic Regression Model	0.751	0.037	0.678-0.824	<0.001	0.761	0.672	0.433
Decision Tree Model	0.709	0.057	0.596-0.821	<0.001	0.616	0.823	0.439
Random Forest Model	0.774	0.049	0.678-0.869	<0.001	0.748	0.71	0.458

**Figure 6:**
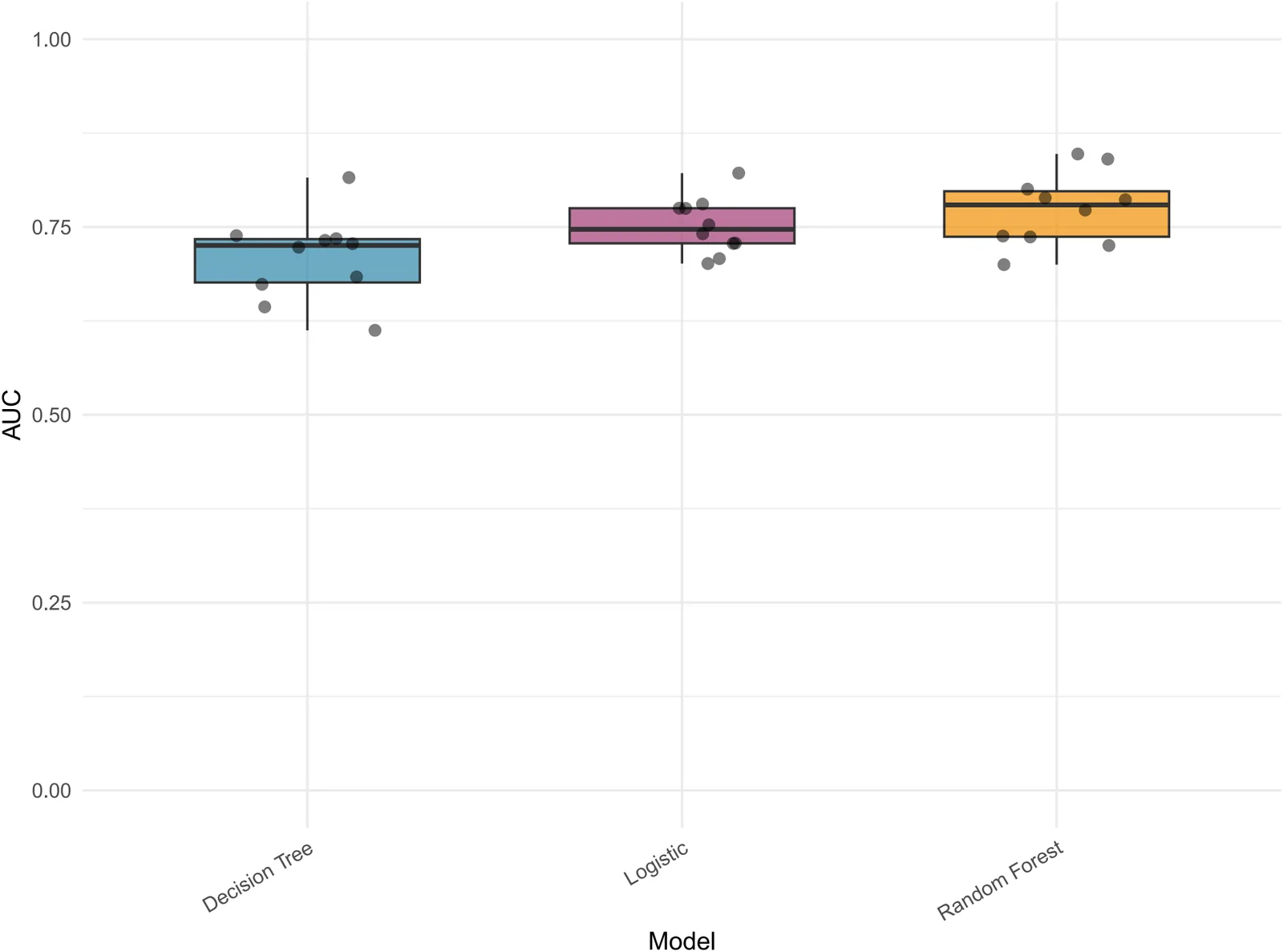
Boxplot of AUC values from 10-fold cross-validation for three prediction models.

DeLong’s tests showed no significant differences in AUC among the three models (RF vs. logistic regression: p = 0.972; RF vs. decision tree: p = 0.752; logistic regression vs. decision tree: p = 0.766) (**Supplementary Table 4**). Across cross-validation folds, the RF model showed a slightly higher median AUC, whereas the decision tree model demonstrated greater variability (**Figure 6**).

**Figure 7:**
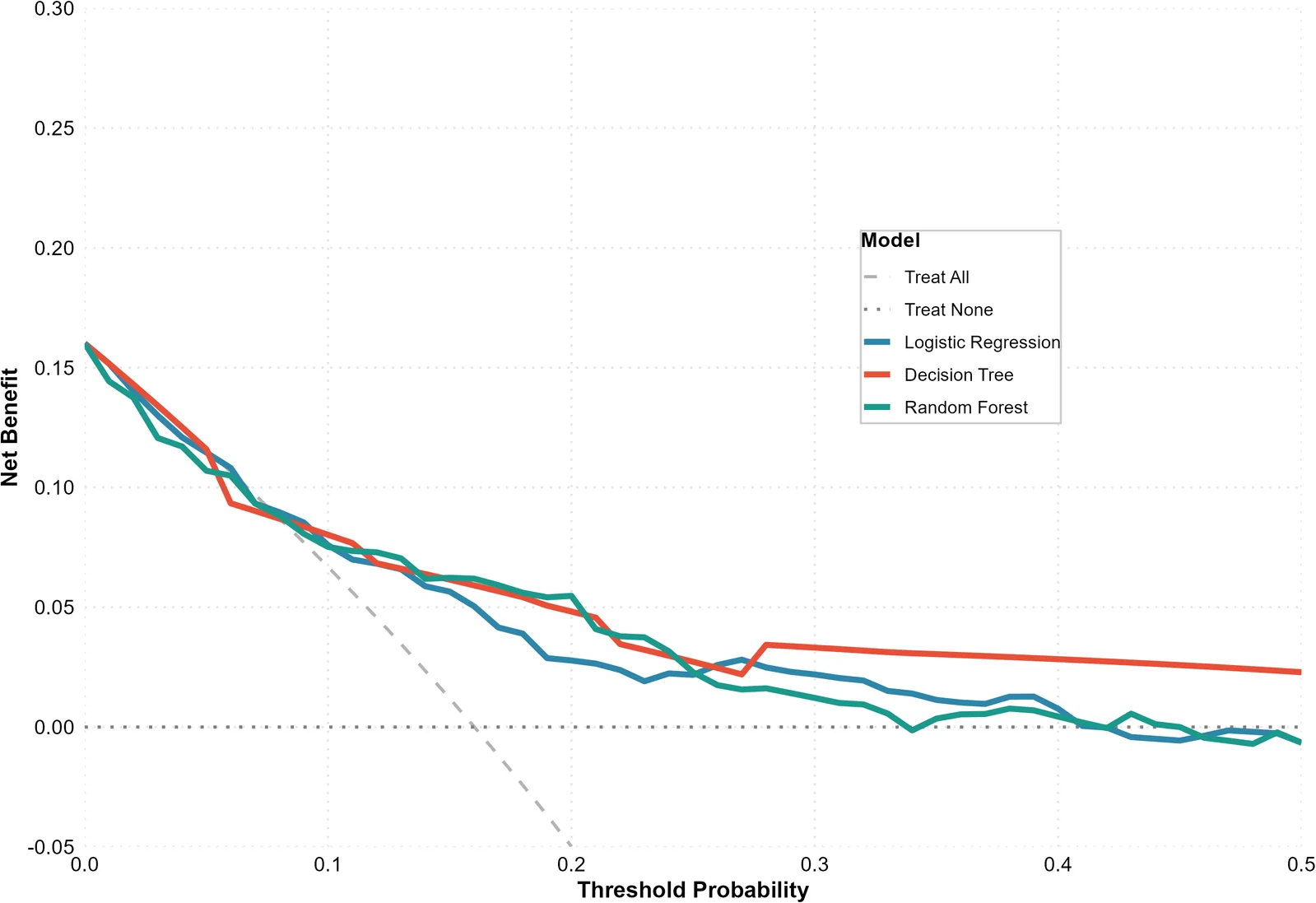
Decision curve analysis of prediction models.

Decision curve analysis indicated that the RF model provided the highest net clinical benefit across most threshold probabilities (**Figure 7**).

**Figure 8:**
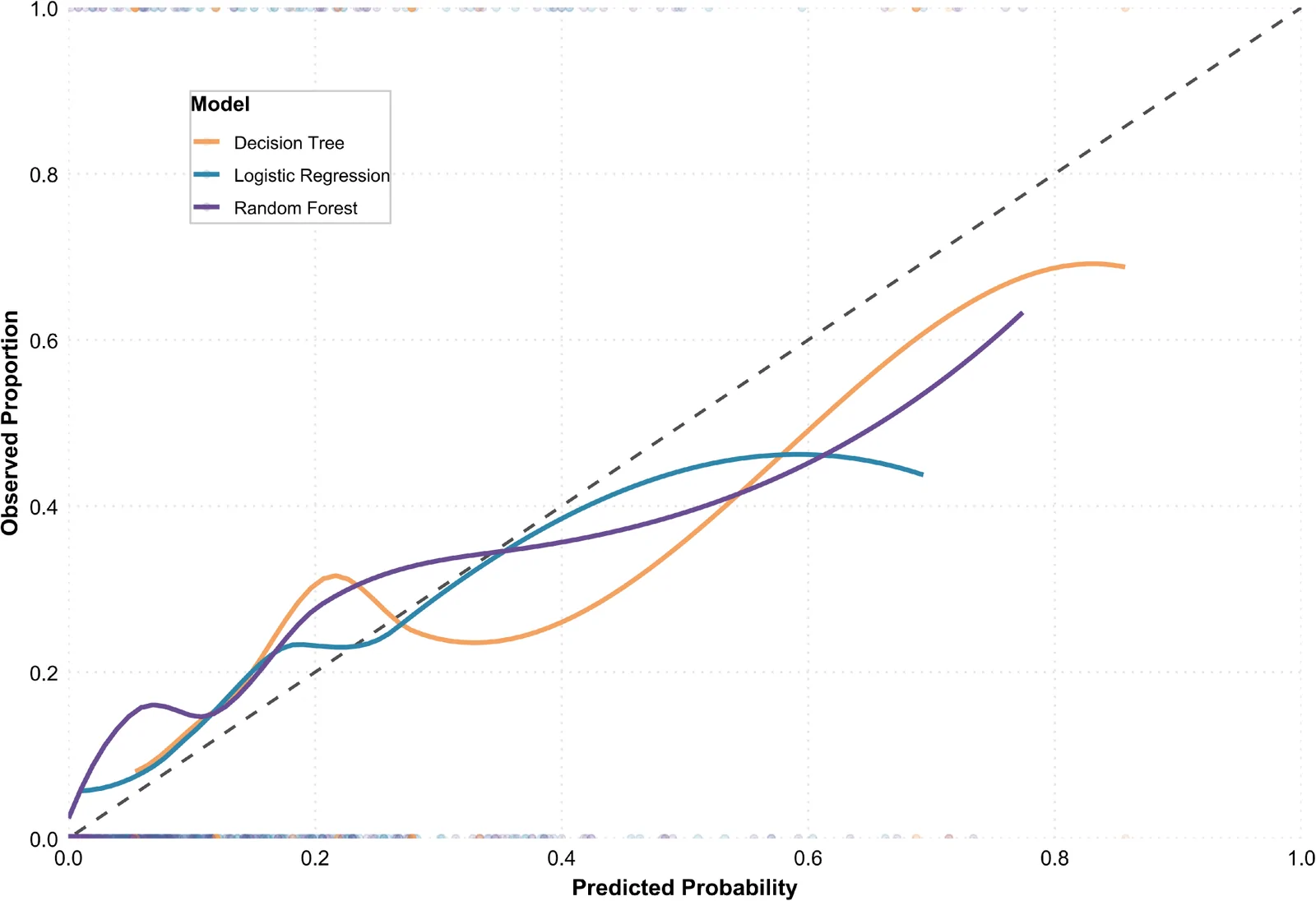
Calibration plots of the three predictive models.

Calibration analysis showed acceptable agreement between predicted and observed probabilities across all models (**Figure 8**). Quantitative calibration metrics suggested that the decision tree model demonstrated the best numerical calibration performance, with the lowest Brier score (0.1142) and calibration intercept (0.02) and slope (0.96) closest to the optimal values. The logistic regression model showed an intercept of 0.10 and slope of 0.88, whereas the RF model showed an intercept of 0.15 and slope of 0.82.

## 4 Discussion

### 4.1 Overall model performance and comparison

To our knowledge, this is the first study to systematically compare traditional logistic regression with machine learning models for predicting diabetic retinopathy (DR) screening adherence in community-dwelling older adults with diabetes while integrating Protection Motivation Theory (PMT)-based psychological constructs.

Among the three models, the random forest (RF) model achieved a marginally higher AUC (0.774), followed by logistic regression (AUC = 0.751) and decision tree (AUC = 0.709). However, DeLong’s tests indicated that these differences were not statistically significant (all p > 0.05). RF demonstrated relatively stable performance under 10-fold cross-validation, suggesting consistent behavior across folds in this complex behavioral dataset.

These findings are consistent with previous evidence indicating that, when methodological bias is minimized, machine learning models may not substantially outperform logistic regression in clinical prediction tasks (27). In the present study, the three models showed comparable discriminative performance, suggesting that the added complexity of ensemble learning may not translate into meaningful improvements in AUC for this specific prediction task. Importantly, model selection in clinical prediction should consider not only discrimination but also interpretability, calibration, and clinical utility (28).

Although the decision tree model showed relatively high specificity (0.823), its sensitivity was lower (0.616) than that of logistic regression and random forest models. This pattern may reflect the intrinsic characteristics of decision tree algorithms, which rely on sequential splitting rules and may produce more conservative classification boundaries. Therefore, the decision tree model may be useful for interpretable risk stratification, particularly for identifying individuals with poor adherence, but may be less suitable as a standalone screening tool when minimizing missed cases is prioritized. In contrast, the random forest model showed a numerically higher AUC and a more balanced sensitivity-specificity profile, though this advantage did not reach statistical significance.

### 4.2 Key predictors and algorithmic interpretation

The reduction from 17 univariable predictors to six independent predictors in multivariable analysis highlights the importance of controlling for confounding and identifying clinically relevant factors. Across the three models, common predictors included HbA1c, self-efficacy, perceived severity, previous ocular disease history, and exercise therapy, although their relative importance varied across algorithms.

The decision tree model identified previous ocular disease history as the root node, whereas the random forest (RF) model ranked HbA1c as the most important predictor, followed by self-efficacy and perceived severity. This difference reflects distinct algorithmic mechanisms: decision trees prioritize variables that provide the greatest immediate improvement in node separation, whereas RF evaluates predictor contribution by aggregating information across multiple trees. Therefore, previous ocular disease history may be useful for identifying specific high-risk subgroups, while HbA1c may represent a more consistent predictor across populations. This finding is consistent with previous evidence highlighting HbA1c as an important determinant of diabetes-related outcomes (29).

Although RF achieved a slightly higher AUC, the difference was not statistically significant. Calibration analysis further showed that the decision tree model had the best quantitative calibration performance, with the lowest Brier score (0.1142), calibration intercept (0.02), and slope (0.96). These findings indicate that model selection should consider multiple performance dimensions, including discrimination, calibration, and clinical utility, rather than AUC alone. RF-based variable importance provides additional insight into the relative contribution of predictors within an ensemble learning framework (30).

The six predictors were used as a unified input set across all models to ensure fair comparison; therefore, variable importance rankings should be interpreted within this framework rather than as independent feature selection. For example, smoking status showed near-zero importance in RF (0.15) but remained significant in logistic regression (OR = 0.554, 95% CI: 0.335–0.888, p = 0.017), reflecting differences in algorithmic learning mechanisms.

### 4.3 Psychological mechanisms and behavioral explanation

Previous ocular disease history was strongly associated with higher screening adherence, consistent with the “personal experience effect,” whereby direct disease exposure may exert a stronger influence on health behaviors than indirect health education (31). This finding is supported by qualitative evidence showing that limited awareness of DR screening is a major barrier, whereas previous ocular experiences may promote healthcare-seeking behavior (32).

Unexpectedly, higher self-efficacy and perceived severity were associated with lower screening adherence, which appears inconsistent with classical Protection Motivation Theory (PMT). These findings should be interpreted cautiously. First, although the PMT-based questionnaire showed good reliability and validity (Cronbach’s α = 0.865; S-CVI = 0.968), it was initially applied in this population, and some items (particularly self-efficacy) assessed general diabetes management confidence rather than task-specific confidence regarding fundus examination attendance. In addition, response style bias and social desirability effects may have influenced psychological assessments.

Second, the cross-sectional design precludes causal interpretation. Individuals with delayed screening or negative healthcare experiences may subsequently develop lower confidence in diabetes-related behaviors, resulting in reverse associations. Third, unmeasured factors, including healthcare access, social support, competing health priorities, and previous healthcare experiences, may partly explain these findings. Cultural interpretation of psychological constructs may also contribute, as perceived severity does not always translate into preventive action.

From a behavioral perspective, higher self-efficacy may reflect confidence in overall diabetes management rather than confidence in completing DR screening. Individuals with greater perceived control over their disease may underestimate the need for regular retinal examinations. Similarly, higher perceived severity may trigger avoidance responses rather than protective behaviors. PMT suggests that threat appraisal and coping appraisal alone may not guarantee behavioral adoption, as response efficacy, perceived response costs, and intention-to-action processes may influence subsequent behaviors (33–35).

Interaction terms between PMT constructs and clinical variables were not included to maintain model parsimony and reduce the risk of overfitting given the limited number of positive adherence events (n = 159). Although tree-based models may capture some nonlinear relationships, explicit interaction testing was not performed. Future studies with larger longitudinal cohorts and refined measurement tools are needed to clarify how PMT constructs interact with clinical characteristics in influencing screening adherence.

### 4.4 Metabolic control, behavioral substitution, and clinical implications

HbA1c emerged as the strongest predictor in the RF model and remained significantly associated with screening adherence in multivariable logistic regression. Poor glycemic control was consistently associated with lower adherence to fundus examinations. Decision tree analysis further identified HbA1c as an important splitting variable, supporting its potential value for clinical risk stratification. Previous guidelines have proposed HbA1c-based thresholds for DR risk assessment (36). The threshold identified in this study (~6.1%) was slightly lower than conventional glycemic targets (7.0–8.0% for older adults), reflecting an algorithmic split point for behavioral prediction rather than a treatment goal. Its interpretation requires consideration of the broader decision tree context, as HbA1c served as a subsequent splitting variable after past ocular history, self-efficacy, and perceived severity. In this community-dwelling population, HbA1c values below 6.1% may identify individuals with better diabetes self-management who nevertheless exhibit poor screening adherence, potentially due to behavioral substitution effects, rather than representing a treatment goal.

Interestingly, exercise therapy showed a negative association with screening adherence. This finding does not imply that exercise reduces screening participation; rather, it may indicate differences in patients’ perceptions and patterns of diabetes self-management. One possible explanation is a behavioral substitution effect, whereby individuals who actively engage in lifestyle modification may perceive exercise as an effective strategy for diabetes control and consequently underestimate the additional importance of preventive screening behaviors (37). Similar patterns have been observed in health behavior research, where engagement in one health-promoting behavior may not necessarily translate into adherence to other recommended preventive practices. However, this interpretation remains hypothetical, and the underlying mechanisms require further investigation using longitudinal studies incorporating measures of health beliefs and self-management behaviors. Clinically, these findings highlight the need to emphasize comprehensive diabetes management, ensuring that lifestyle interventions are integrated with, rather than substituted for, recommended DR screening practices.

### 4.5 Clinical translation and decision curve analysis

This study integrates sociodemographic, clinical, behavioral, and PMT-based psychological variables into machine learning models for DR screening adherence, supporting a multidimensional approach to risk prediction. From a clinical perspective, RF demonstrated a marginally higher AUC, though this difference was not statistically significant, whereas logistic regression provided greater interpretability and decision tree models offered simplified rule-based stratification.

Decision curve analysis (DCA) further supported the clinical usefulness of the models. Across clinically relevant threshold probabilities, RF provided the highest net benefit compared with both logistic regression and decision tree models, providing a different, alternative clinical decision-making approach for identifying patients at risk of poor adherence.

From a practical standpoint, decision tree-derived rules may facilitate simplified screening pathways. Patients without prior ocular disease history, with low self-efficacy, and elevated HbA1c may represent a high-risk subgroup requiring targeted intervention.The decision tree model’s lower sensitivity (0.616) but higher specificity (0.823) may be particularly useful in resource-limited settings where minimizing false-positive referrals is prioritized. However, the increased risk of missed cases (false negatives) is concerning for DR screening, as delayed detection can lead to preventable vision loss. Therefore, the decision tree model should be viewed as a supplementary triage tool rather than a standalone screening instrument. Further external validation and cost-effectiveness analyses are needed before its clinical implementation in these settings.

Current diabetes management guidelines emphasize integrated long-term care strategies combining metabolic control and behavioral interventions to improve screening adherence (38). Our findings further support multidimensional intervention strategies targeting both clinical and psychological determinants.Future studies may further explore advanced artificial intelligence approaches, including explainable deep learning and multimodal models, to integrate diverse healthcare data sources and enhance personalized healthcare decision support.

## 5 Limitations

This study has several limitations. First, the cross-sectional design limits causal inference. In addition, PMT-based psychological constructs were assessed at questionnaire administration, whereas screening adherence was retrospectively assessed over the preceding 12 months; therefore, these perceptions may not fully represent those at the time of screening decisions. Future prospective studies should assess PMT constructs before screening periods.

Second, all participants were recruited from a single geographic region (Nantong, China), which may limit generalizability. Since PMT-based perceptions may vary across cultural backgrounds, health beliefs, and healthcare systems, multicenter studies involving diverse populations and settings are needed to further validate the models.

Third, screening adherence was based on self-reported data, which may introduce recall and reporting bias. Future studies incorporating electronic health records or healthcare databases may provide more objective adherence measures. Additionally, future research should consider incorporating validated social desirability scales (e.g., the Marlowe–Crowne Social Desirability Scale) or using indirect questioning techniques to further assess and control for this potential bias.

Fourth, although stratified 10-fold cross-validation was applied, independent external validation and additional resampling-based validation approaches were not performed. Future studies incorporating external validation cohorts, bootstrap-based methods, and nested validation procedures are needed to further evaluate model robustness. Incorporating DR severity grading may also facilitate the development of more clinically tailored prediction models.

## 6 Conclusion

In conclusion, this study demonstrates that machine learning models demonstrated comparable discriminative performance to traditional logistic regression for predicting the risk of poor adherence to diabetic retinopathy screening among community-dwelling older adults with diabetes, while offering distinct profiles in calibration and clinical net benefit. Previous ocular disease history, HbA1c, perceived severity, self-efficacy, and exercise therapy were identified as key predictors. Notably, the negative associations of self-efficacy and exercise therapy suggest a distinct psychological and cognitive pattern in this population, potentially reflecting a cognitive-behavioral disconnect that leads patients to underestimate the necessity of routine fundus screening. These findings provide evidence for the development of multidimensional risk assessment tools that integrate psychological, clinical, and behavioral factors, and support the implementation of precision stratified interventions targeting cognitive-behavioral gaps in DR screening adherence.

## Data Availability

The raw data supporting the conclusions of this article will be made available by the authors, without undue reservation.
