# Correction: Machine learning versus traditional regression models for predicting diabetic retinopathy screening adherence among community-dwelling older adults with diabetes

**DOI:** 10.3389/fendo.2026.1958085

**Published:** 2026-08-12

**Authors:** Frontiers Production Office

**Affiliations:** Frontiers Media SA, Lausanne, Switzerland

**Keywords:** community-dwelling older adults, diabetic retinopathy screening adherence, logistic regression, machine learning, protection motivation theory, random forest

There was a mistake in **Figures 1**–**4** as published. The image used in **Figure 1** should have been **Figure 3**; the image used in **Figure 2** should have been **Figure 4**; the image used in **Figure 3** should have been **Figure 1**; and the image used in **Figure 4** should have been **Figure 2**. The figure captions in the published article were correct and have not been updated. The corrected figures appear below.

**Figure 1:**
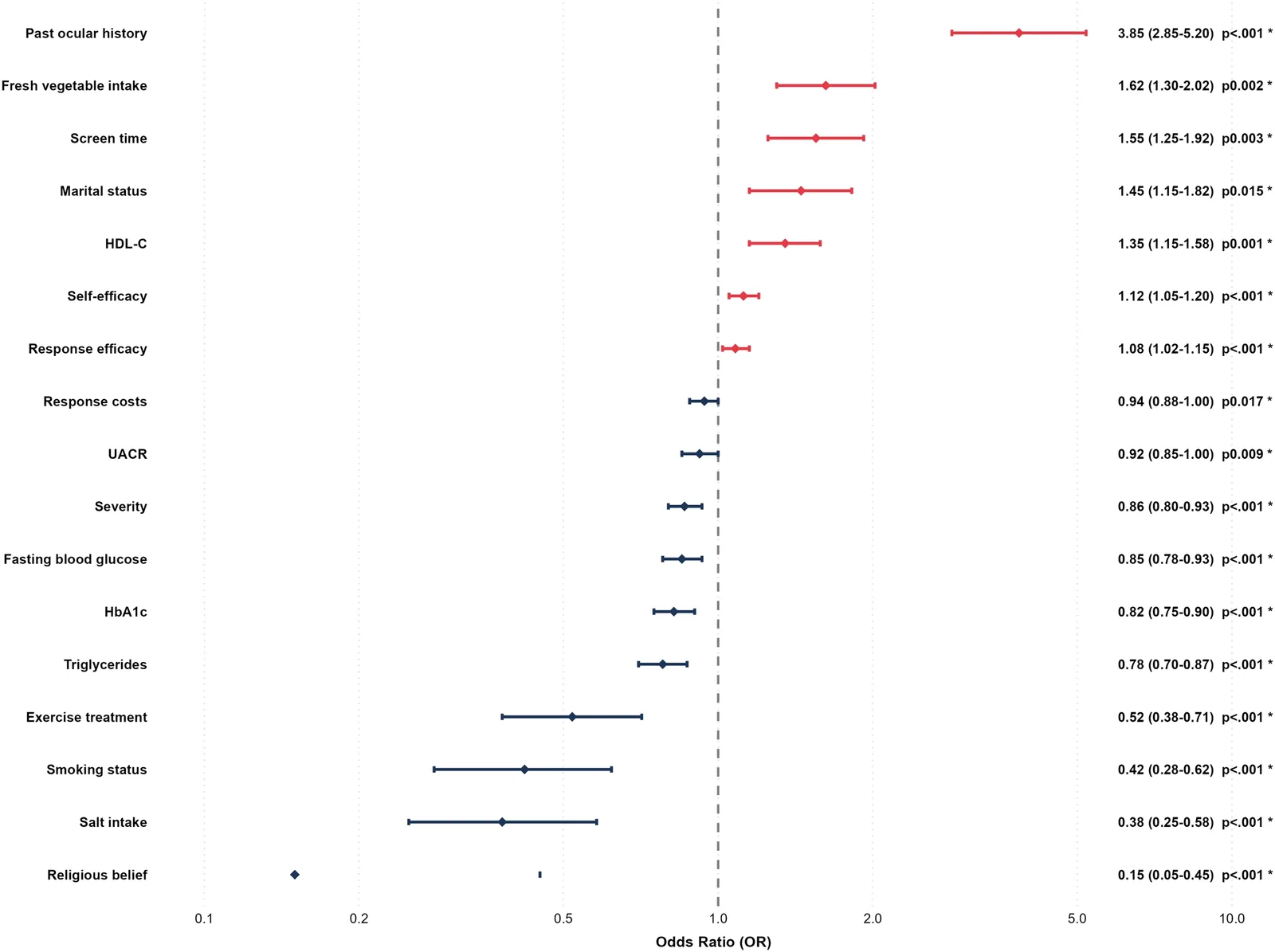
Forest plot of univariable logistic regression analysis.

**Figure 2:**
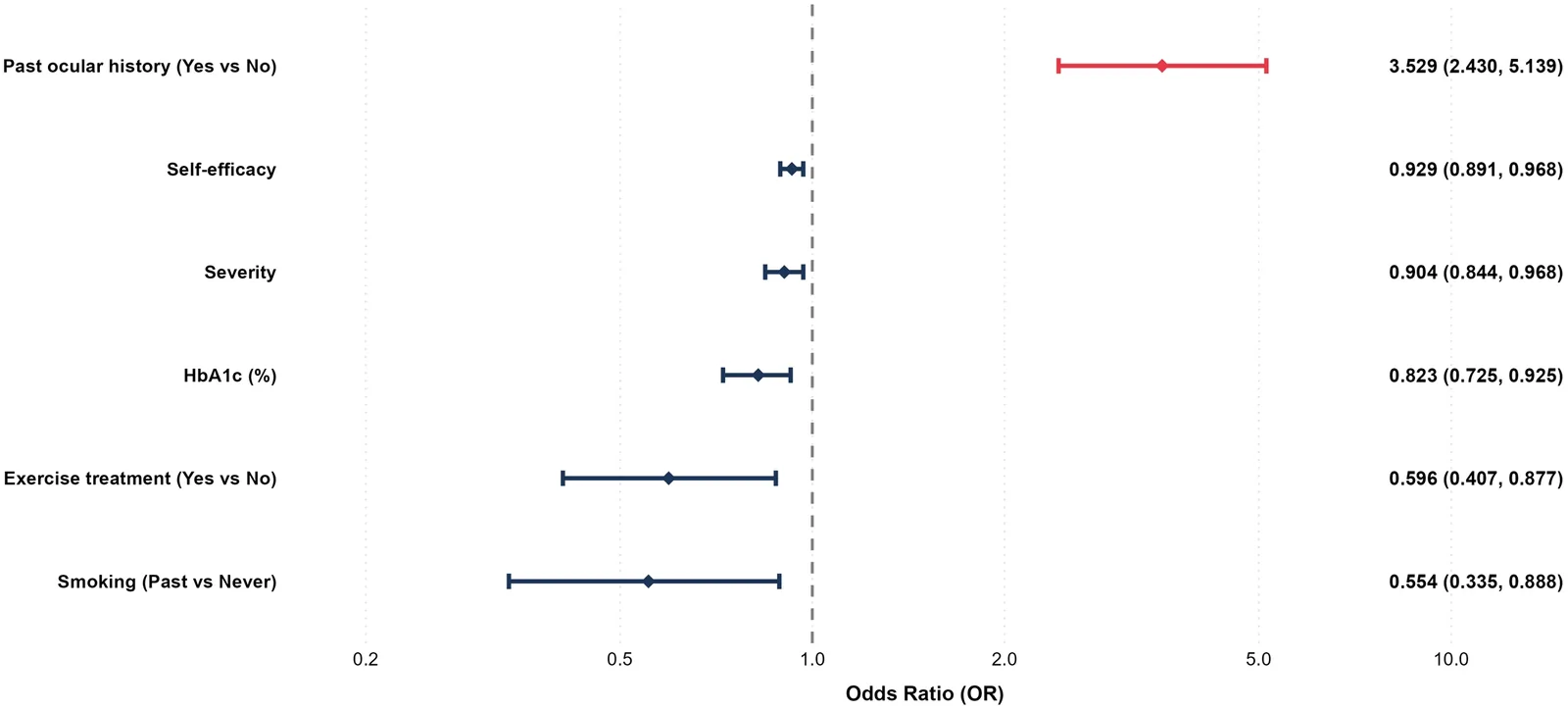
Forest plot of multivariable logistic regression analysis.

**Figure 3:**
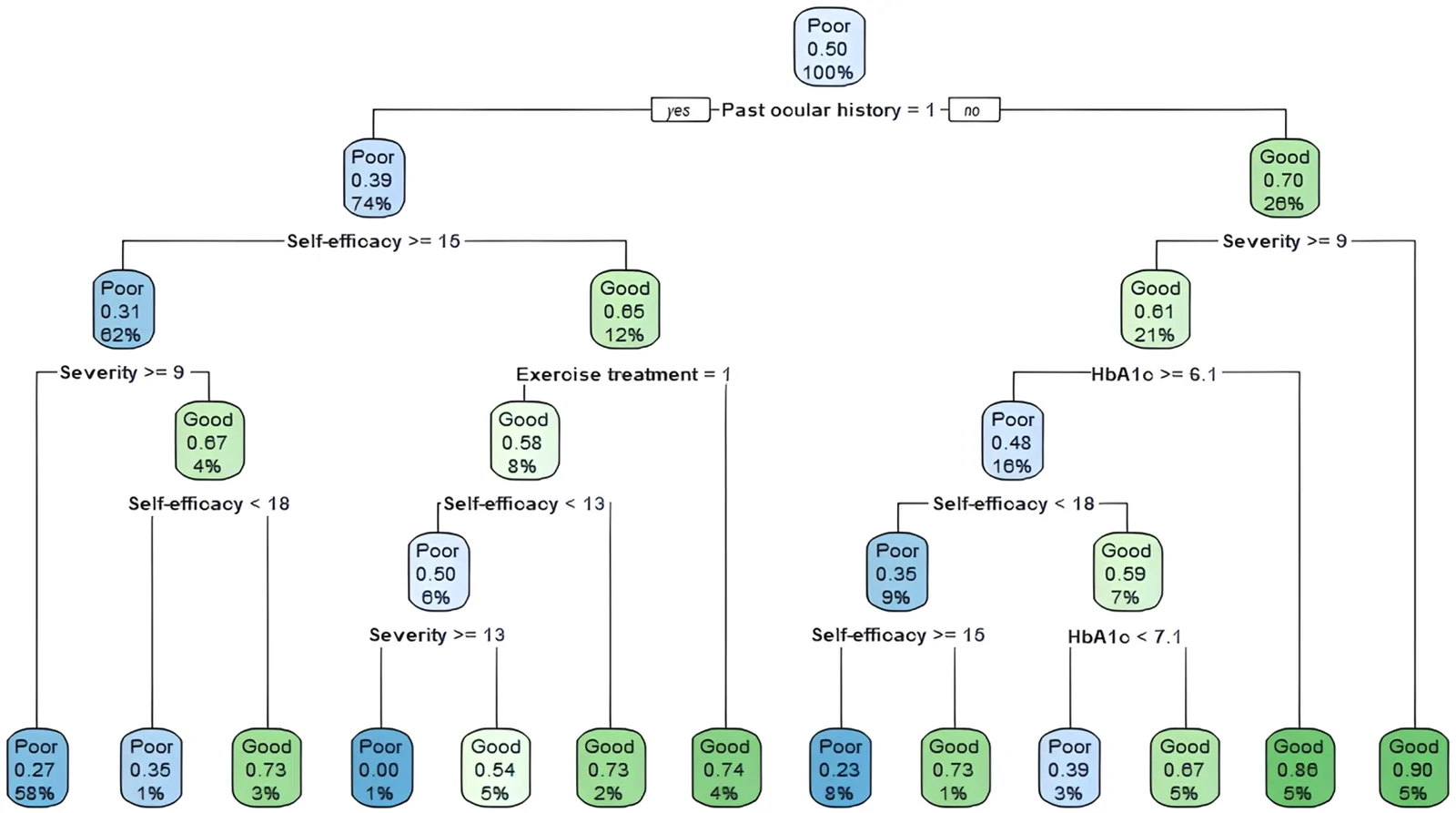
Machine learning decision tree model for fundus examination adherence.

**Figure 4:**
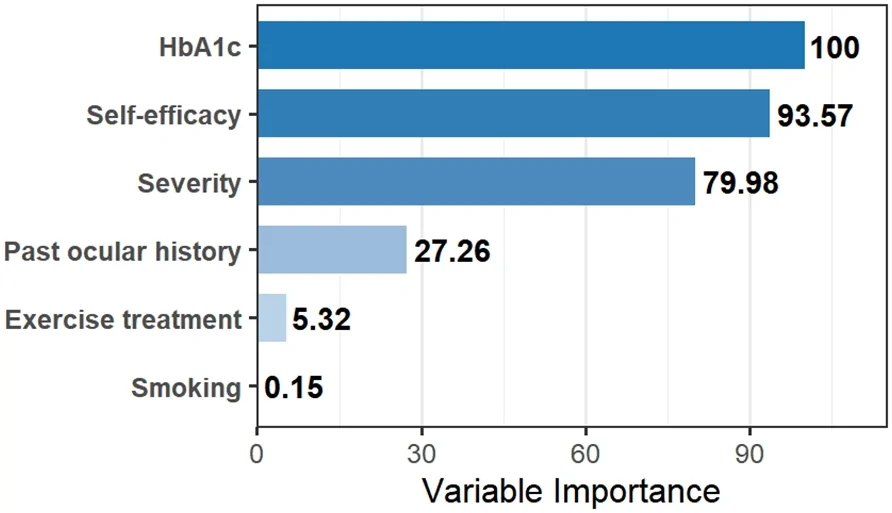
Variable importance for fundus examination adherence prediction.

The original version of this article has been updated.

