# Correction: Machine learning versus traditional regression models for predicting diabetic retinopathy screening adherence among community-dwelling older adults with diabetes

**DOI:** 10.3389/fendo.2026.1966845

**Published:** 2026-08-24

**Authors:** Yawen Wang, Lanting Xia, Guiling Geng, Chunxing Ge, Haiyan Shen, Xiangyun Qian

**Affiliations:** 1 School of Nursing and Rehabilitation, Nantong University, Nantong, China; 2 Teaching and Research Section, Nantong College of Science and Technology, Nantong, China; 3 Department of Geriatrics, Nantong Third People's Hospital (Affiliated Nantong Hospital 3 of Nantong University), Nantong, China; 4 Nursing Department, Nantong Third People's Hospital (Affiliated Nantong Hospital 3 of Nantong University), Nantong, China

**Keywords:** community-dwelling older adults, diabetic retinopathy screening adherence, logistic regression, machine learning, protection motivation theory, random forest

The order of authors in the author list of the published paper was erroneously given as:

“Yawen Wang¹†, Lanting Xia¹†, Guiling Geng², Chunxing Ge¹, Xiangyun Qian³* and Haiyan Shen^4^*”

The correct author list reads:

“Yawen Wang¹†,” Lanting Xia¹†, Guiling Geng², Chunxing Ge¹, Haiyan Shen³* and Xiangyun Qian^4^*

The original version of this article has been updated.

