# Supplementary material for: Machine learning versus traditional regression models for predicting diabetic retinopathy screening adherence among community-dwelling older adults with diabetes
Source: Front Endocrinol (Lausanne). 2026 Aug 4;17:1909594. doi: 10.3389/fendo.2026.1909594 (PMC13494464; doi:10.3389/fendo.2026.1909594)
Supplement: Supplementary file 1 [file Table1.docx]

# **Supplementary Table1** PMT-Based Diabetic Retinopathy Risk Perception Questionnaire

**Note:** This questionnaire was originally developed in Chinese and administered to all participants in Chinese. The English version was prepared using a standard forward-backward translation procedure to ensure semantic equivalence. Responses were scored on a 5-point Likert scale (1 = Strongly disagree, 2 = Disagree, 3 = Uncertain, 4 = Agree, 5 = Strongly agree). Items 10–12 (Internal Rewards), 13–15 (External Rewards), 27–30 (Response Cost) were reverse-scored prior to analysis so that higher scores consistently indicated greater perceived risk of diabetic retinopathy.

| **Dimension** | **ItemNo.** | **EnglishItem** | **ChineseOriginal** |
| --- | --- | --- | --- |
| A. Perceived Severity | 1 | I know that diabetes may lead to vision loss or even blindness. | 我知道糖尿病可能会导致视力下降甚至失明。 |
| A. Perceived Severity | 2 | Vision loss affects daily activities such as reading, driving, recognizing objects, and walking. | 视力下降会影响日常生活，如阅读、驾车、识物、行走等。 |
| A. Perceived Severity | 3 | Vision loss or blindness would increase family financial and caregiving burden. | 视力下降甚至失明会加重家庭经济和照护负担。 |
| B. Perceived Susceptibility | 4 | If I do not pay attention to controlling my blood glucose, it may increase the risk of vision loss or even blindness. | 如果我不注意控制血糖，可能会增加视力下降甚至失明的风险。 |
| B. Perceived Susceptibility | 5 | If I do not control hypertension, hyperlipidemia, or kidney disease and other complications well, my risk of vision loss or even blindness caused by diabetes will increase. | 如果我控制不好高血压、高血脂或者肾脏病等并发症，我患糖尿病引起的视力下降甚至失明的风险会增加。 |
| B. Perceived Susceptibility | 6 | If I do not follow medical advice for treatment or fundus examination re-check, it will increase the risk of vision loss or even blindness. | 如果我不按照医嘱治疗或复查眼底，会增加视力下降甚至失明的风险。 |
| B. Perceived Susceptibility | 7 | If I do not use my eyes properly for a long time, it will increase the risk of vision loss or even blindness caused by diabetes. | 如果我长期不规范用眼，会增加糖尿病引起的视力下降甚至失明的风险。 |
| B. Perceived Susceptibility | 8 | The longer I have had diabetes, the more likely I am to increase the risk of vision loss or even blindness caused by diabetes. | 我患糖尿病的时间越长，越容易增加糖尿病引起的视力下降甚至失明的风险。 |
| B. Perceived Susceptibility | 9 | If I have unhealthy behavioral habits (smoking, excessive drinking, etc.), it will increase the risk of vision loss or even blindness. | 如果我有不健康的行为习惯（吸烟、过度饮酒等），会增加视力下降甚至失明的风险。 |
| C. Internal Rewards | 10 | Irregular medication makes me feel relaxed and comfortable. | 不规律服药让我感到很轻松、自在。 |
| C. Internal Rewards | 11 | I think an uncontrolled life makes me comfortable, such as not controlling diet, drinking, or smoking. | 我觉得不受控制的生活让我很舒服，如不控制饮食、喝酒、吸烟等。 |
| C. Internal Rewards | 12 | Reducing or avoiding blood glucose monitoring can reduce my pain (or trouble) and economic costs. | 不测或减少血糖监测能减少我的痛苦（或麻烦）和经济费用。 |
| D. External Rewards | 13 | People around me believe that diabetes will not cause vision problems. | 我身边的人认为糖尿病不会引起视力问题。 |
| D. External Rewards | 14 | Diabetic patients around me do not undergo fundus examination on time, and their vision has not had problems. | 我身边的糖尿病患者不按时进行眼底检查，视力没有出现问题。 |
| D. External Rewards | 15 | People around me believe that unhealthy lifestyles will not lead to vision problems caused by diabetes. | 我身边的人认为不健康的生活方式不会导致糖尿病引发的视力问题。 |
| E. Self-Efficacy | 16 | I am confident that I can control my blood glucose within the normal range. | 我有信心将血糖控制在正常范围。 |
| E. Self-Efficacy | 17 | I am confident that I can improve my lifestyle to control risk factors such as blood pressure and blood lipids that aggravate diabetes complications. | 我有信心通过改善生活方式控制血压、血脂等加重糖尿病等并发症危险因素。 |
| E. Self-Efficacy | 18 | I am confident that I can persist in regular fundus examination re-checks. | 我有信心坚持定期复查眼底。 |
| E. Self-Efficacy | 19 | I am confident that I can persist in proper eye use. | 我有信心坚持规范用眼。 |
| E. Self-Efficacy | 20 | I am confident that I can persist in a healthy lifestyle. | 我有信心坚持健康的生活方式。 |
| E. Self-Efficacy | 21 | I am confident that I can persist in following medical advice for early prevention or treatment of eye diseases. | 我有信心坚持遵医嘱进行眼睛的早期预防或治疗。 |
| F. Response Efficacy | 22 | I believe that monitoring and controlling blood glucose can reduce my risk of vision loss or even blindness caused by diabetes. | 我觉得监测及控制血糖，能减少我糖尿病引起的视力下降甚至失明的风险。 |
| F. Response Efficacy | 23 | I believe that a healthy lifestyle will reduce vision problems caused by diabetes. | 我觉得健康的生活方式，会减少糖尿病引起的视力问题。 |
| F. Response Efficacy | 24 | I believe that regular fundus examination re-checks can timely detect the risk of vision loss. | 我觉得定期复查眼底，能及时发现视力下降的风险。 |
| F. Response Efficacy | 25 | I believe that proper eye use can reduce the risk of vision loss. | 我觉得规范用眼，能降低视力下降的风险。 |
| F. Response Efficacy | 26 | I believe that regularly receiving diabetes retinopathy-related health education can help me timely detect vision problems. | 我觉得定期接受糖尿病视网膜病相关知识的科普能让我及时发现视力问题。 |
| G. Response Cost | 27 | I believe that a healthy lifestyle will reduce my life pleasure. | 我觉得健康的生活方式会减少我的生活乐趣。 |
| G. Response Cost | 28 | I think regular blood glucose monitoring is troublesome. | 我觉得定期测血糖很麻烦。 |
| G. Response Cost | 29 | I think regular fundus examination re-checks are time-consuming and costly. | 我觉得定期复查眼底很费时、费钱。 |
| G. Response Cost | 30 | I think correct and regular medication will increase my burden. | 我觉得正确规律用药会增加我的负担。 |
