# Supplementary material for: Machine learning versus traditional regression models for predicting diabetic retinopathy screening adherence among community-dwelling older adults with diabetes
Source: Front Endocrinol (Lausanne). 2026 Aug 4;17:1909594. doi: 10.3389/fendo.2026.1909594 (PMC13494464; doi:10.3389/fendo.2026.1909594)
Supplement: Supplementary file 2 [file Table2.docx]

**Supplementary Table 2** Sensitivity analysis using an alternative definition of fundus examination adherence

| **Model** | **AUC (≥2 examinations/year)** | **AUC (≥1 examination/year)** | **Absolute Difference** |
| --- | --- | --- | --- |
| Logistic regression | 0.751 | 0.750 | 0.001 |
| Decision tree | 0.709 | 0.711 | 0.002 |
| Random forest | 0.774 | 0.773 | 0.001 |
