# Supplementary material for: Machine learning versus traditional regression models for predicting diabetic retinopathy screening adherence among community-dwelling older adults with diabetes
Source: Front Endocrinol (Lausanne). 2026 Aug 4;17:1909594. doi: 10.3389/fendo.2026.1909594 (PMC13494464; doi:10.3389/fendo.2026.1909594)
Supplement: Supplementary file 3 [file Table3.docx]

**Supplementary Table 3** Performance metrics of decision tree, logistic regression, and random forest models under different class imbalance-handling strategies

| **Model** | **Strategy** | **AUC** | **Sensitivity** | **Specificity** | **Precision** | **NPV** | **Brier** |
| --- | --- | --- | --- | --- | --- | --- | --- |
| Logistic Regression | No imbalance handling | 0.762 | 0.979 | 0.138 | 0.86 | 0.529 | 0.115 |
|  | SMOTE only | 0.762 | 0.703 | 0.674 | 0.921 | 0.296 | 0.194 |
|  | Class weighting only | 0.762 | 0.696 | 0.674 | 0.92 | 0.293 | 0.198 |
|  | SMOTE + Class weighting | 0.758 | 0.761 | 0.672 | 0.922 | 0.296 | 0.196 |
| Decision Tree | No imbalance handling | 0.69 | 0.962 | 0.258 | 0.876 | 0.548 | 0.119 |
|  | SMOTE only | 0.706 | 0.767 | 0.603 | 0.913 | 0.329 | 0.19 |
|  | Class weighting only | 0.718 | 0.755 | 0.673 | 0.926 | 0.345 | 0.195 |
|  | SMOTE + Class weighting | 0.747 | 0.616 | 0.823 | 0.913 | 0.318 | 0.194 |
| Random Forest | No imbalance handling | 0.78 | 0.97 | 0.213 | 0.87 | 0.572 | 0.113 |
|  | SMOTE only | 0.77 | 0.92 | 0.35 | 0.88 | 0.35 | 0.135 |
|  | Class weighting only | 0.725 | 0.906 | 0.334 | 0.881 | 0.407 | 0.141 |
|  | SMOTE + Class weighting | 0.774 | 0.748 | 0.71 | 0.889 | 0.369 | 0.136 |
