# Supplementary material for: Machine learning versus traditional regression models for predicting diabetic retinopathy screening adherence among community-dwelling older adults with diabetes
Source: Front Endocrinol (Lausanne). 2026 Aug 4;17:1909594. doi: 10.3389/fendo.2026.1909594 (PMC13494464; doi:10.3389/fendo.2026.1909594)
Supplement: Supplementary file 4 [file Table4.docx]

**Supplementary Table 4** DeLong's test for pairwise AUC comparison among the three models

| **Comparison** | **Model 1 AUC** | **Model 2 AUC** | **AUC Difference** | **Z statistic** | **p-value** |
| --- | --- | --- | --- | --- | --- |
| Random Forest vs Logistic | 0.774 | 0.758 | 0.016 | 0.035 | 0.972 |
| Random Forest vs Decision Tree | 0.774 | 0.747 | 0.027 | 0.315 | 0.752 |
| Logistic vs Decision Tree | 0.758 | 0.747 | 0.011 | 0.298 | 0.766 |
